# Supplementary figures and images for: Participatory modelling for poverty alleviation using fuzzy cognitive maps and OWA learning aggregation
Source: PLoS One. 2020 Jun 8;15(6):e0233984. doi: 10.1371/journal.pone.0233984 (PMC7279611; doi:10.1371/journal.pone.0233984)

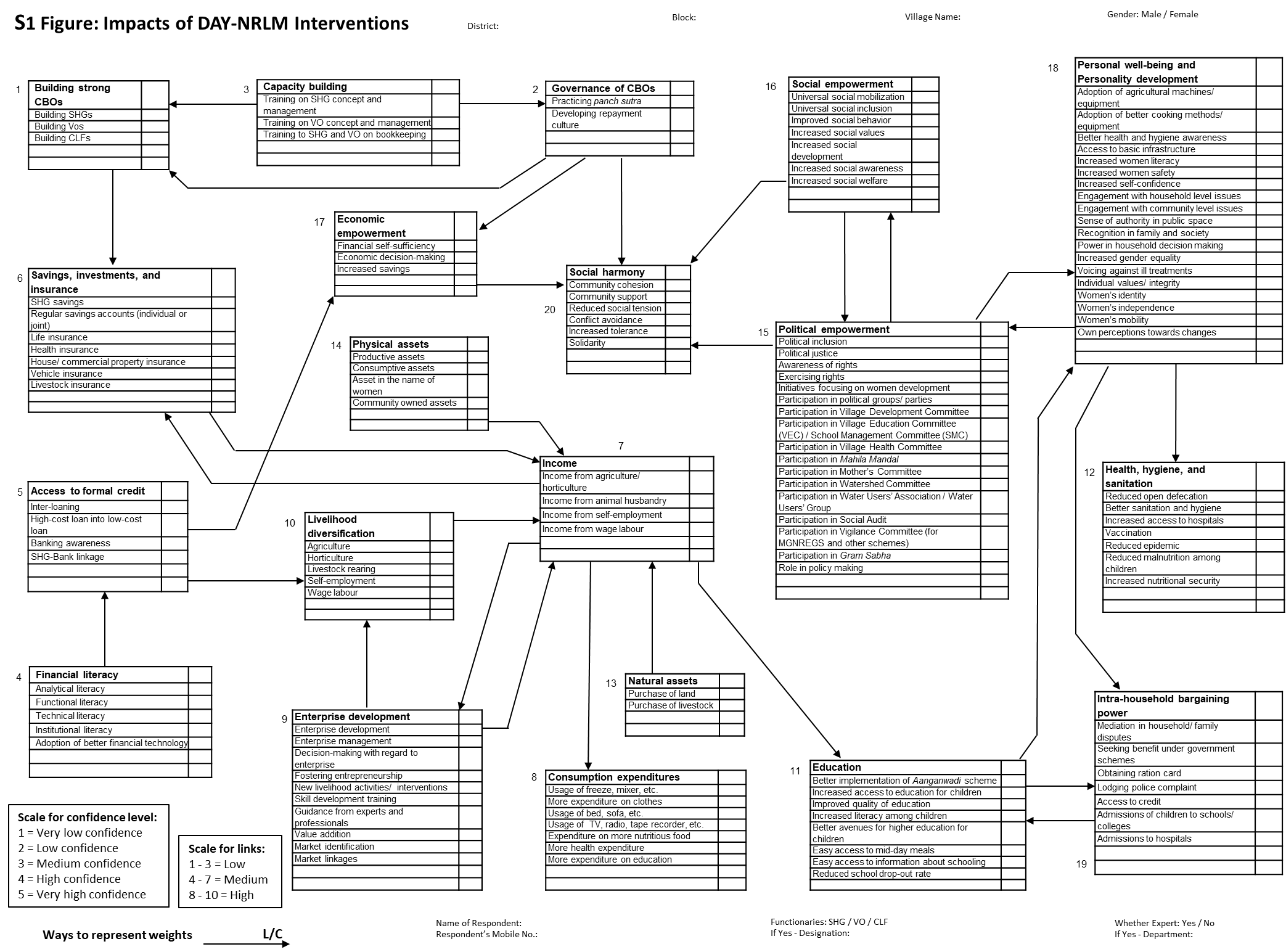

Supplement: S1 Fig — (TIF) [file pone.0233984.s001.tif]

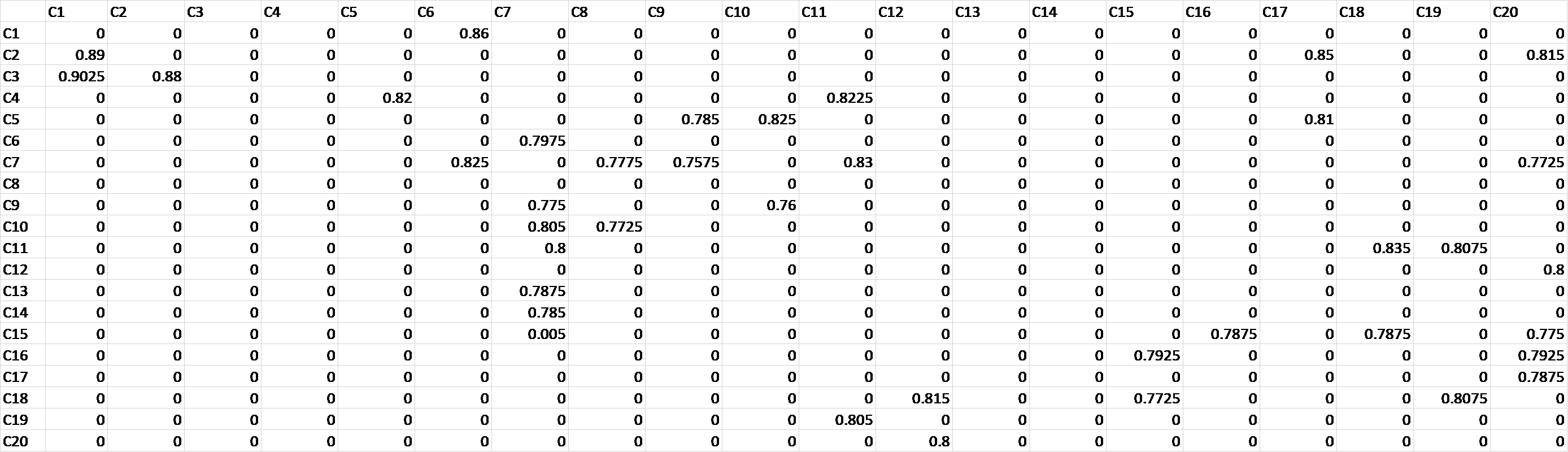

Supplement: S2 Fig — (TIF) [file pone.0233984.s002.tif]

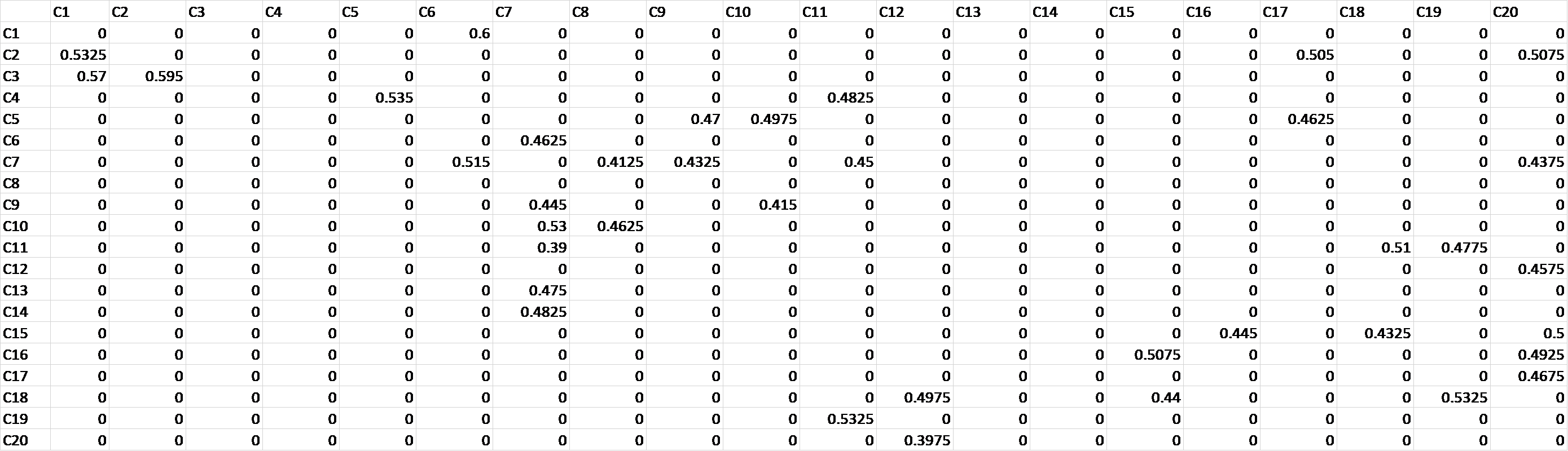

Supplement: S3 Fig — (TIF) [file pone.0233984.s003.tif]

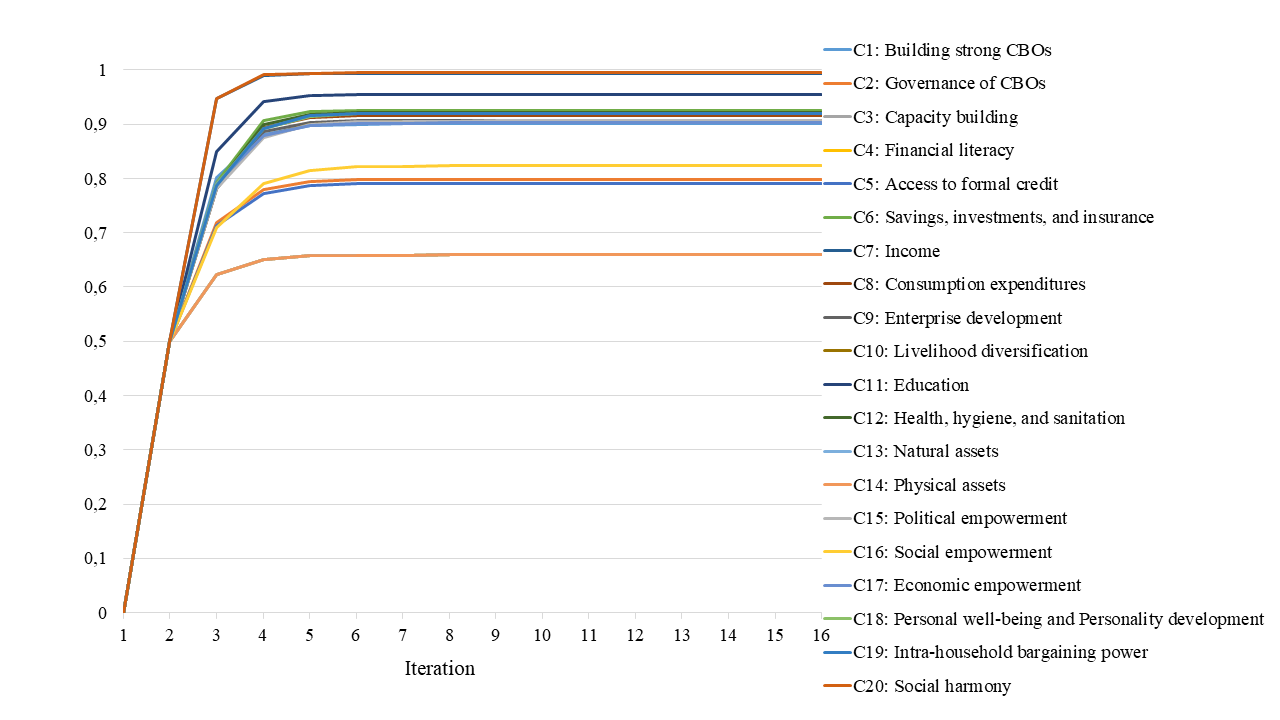

Supplement: S4 Fig — (TIF) [file pone.0233984.s004.tif]

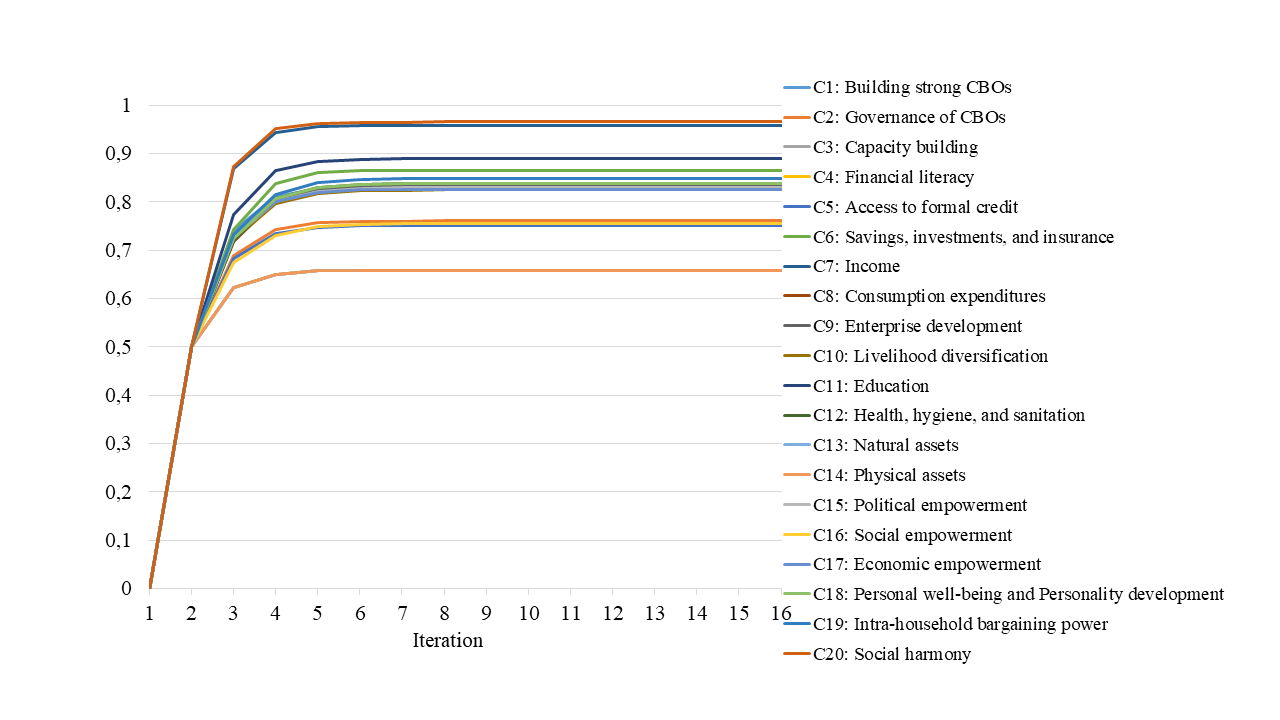

Supplement: S5 Fig — (TIF) [file pone.0233984.s005.tif]
