# Supplementary material for: Participatory modelling for poverty alleviation using fuzzy cognitive maps and OWA learning aggregation
Source: PLoS One. 2020 Jun 8;15(6):e0233984. doi: 10.1371/journal.pone.0233984 (PMC7279611; doi:10.1371/journal.pone.0233984)
Supplement: S1 Table — (DOCX) [file pone.0233984.s006.docx]

**S1 Table. Scenario results (initial and final value) for each concept, for the Average-FCM (L)**

|  | **Scenario 1** | | **Scenario 2** | | **Scenario 3** | | **Scenario 4** | | **Scenario 5** | | | **Scenario 6** | | | **Scenario 7** | | | **Scenario 8** | | | **Scenario 9** | | |
| --- | --- | --- | --- | --- | --- | --- | --- | --- | --- | --- | --- | --- | --- | --- | --- | --- | --- | --- | --- | --- | --- | --- | --- |
| **Key Concept** | **Initial value** | **Final value** | **Initial value** | **Final value** | **Initial value** | **Final value** | **Initial value** | **Final value** | | **Initial value** | **Final value** | **Initial value** | **Final value** | **Initial value** | | **Final value** | **Initial value** | | **Final value** | **Initial value** | | **Final value** |  |
| **C1** | 1 | 1 | 0 | 0.929 | 0 | 0.900 | 0 | 0.900 | | 1 | 1 | 1 | 1 | 1 | | 1 | 0 | | 0.900 | 1 | | 1 |  |
| **C2** | 1 | 1 | 0 | 0.849 | 0 | 0.798 | 0 | 0.798 | | 1 | 1 | 1 | 1 | 1 | | 1 | 0 | | 0.798 | 1 | | 1 |  |
| **C3** | 0 | 0.659 | 1 | 1 | 0 | 0.659 | 0 | 0.659 | | 1 | 1 | 0 | 0.659 | 0 | | 0.659 | 0 | | 0.659 | 1 | | 1 |  |
| **C4** | 0 | 0.659 | 0 | 0.659 | 0 | 0.659 | 0 | 0.659 | | 0 | 0.659 | 0 | 0.659 | 0 | | 0.659 | 0 | | 0.659 | 0 | | 0.659 |  |
| **C5** | 0 | 0.791 | 0 | 0.791 | 1 |  | 0 | 0.791 | | 0 | 0.791 | 1 | 1 | 0 | | 0.791 | 1 | | 1 | 1 | | 1 |  |
| **C6** | 0 | 0.931 | 0 | 0.927 | 0 | 0.925 | 0 | 0.925 | | 0 | 0.931 | 0 | 0.931 | 0 | | 0.932 | 0 | | 0.925 | 0 | | 0.932 |  |
| **C7** | 0 | 0.993 | 0 | 0.993 | 0 | 0.993 | 0 | 0.994 | | 0 | 0.993 | 0 | 0.993 | 0 | | 0.994 | 0 | | 0.994 | 0 | | 0.994 |  |
| **C8** | 0 | 0.915 | 0 | 0.915 | 0 | 0.916 | 0 | 0.921 | | 0 | 0.915 | 0 | 0.916 | 0 | | 0.921 | 0 | | 0.921 | 0 | | 0.921 |  |
| **C9** | 0 | 0.907 | 0 | 0.907 | 0 | 0.921 | 1 | 1 | | 0 | 0.907 | 0 | 0.921 | 1 | | 1 | 1 | | 1 | 1 | | 1 |  |
| **C10** | 0 | 0.904 | 0 | 0.904 | 0 | 0.920 | 1 | 1 | | 0 | 0.904 | 0 | 0.920 | 1 | | 1 | 1 | | 1 | 1 | | 1 |  |
| **C11** | 0 | 0.955 | 0 | 0.955 | 0 | 0.955 | 0 | 0.955 | | 0 | 0.955 | 0 | 0.955 | 0 | | 0.955 | 0 | | 0.955 | 0 | | 0.955 |  |
| **C12** | 0 | 0.922 | 0 | 0.922 | 0 | 0.922 | 0 | 0.922 | | 0 | 0.922 | 0 | 0.922 | 0 | | 0.922 | 0 | | 0.922 | 0 | | 0.922 |  |
| **C13** | 0 | 0.659 | 0 | 0.659 | 0 | 0.659 | 0 | 0.659 | | 0 | 0.659 | 0 | 0.659 | 0 | | 0.659 | 0 | | 0.659 | 0 | | 0.659 |  |
| **C14** | 0 | 0.659 | 0 | 0.659 | 0 | 0.659 | 0 | 0.659 | | 0 | 0.659 | 0 | 0.659 | 0 | | 0.659 | 0 | | 0.659 | 0 | | 0.659 |  |
| **C15** | 0 | 0.905 | 0 | 0.905 | 0 | 0.905 | 0 | 0.905 | | 0 | 0.905 | 0 | 0.905 | 0 | | 0.905 | 0 | | 0.905 | 0 | | 0.905 |  |
| **C16** | 0 | 0.823 | 0 | 0.823 | 0 | 0.823 | 0 | 0.823 | | 0 | 0.823 | 0 | 0.823 | 0 | | 0.823 | 0 | | 0.823 | 0 | | 0.823 |  |
| **C17** | 0 | 0.917 | 0 | 0.906 | 0 | 0.917 | 0 | 0.902 | | 0 | 0.917 | 0 | 0.930 | 0 | | 0.917 | 0 | | 0.917 | 0 | | 0.930 |  |
| **C18** | 0 | 0.919 | 0 | 0.919 | 0 | 0.919 | 0 | 0.919 | | 0 | 0.919 | 0 | 0.919 | 0 | | 0.919 | 0 | | 0.919 | 0 | | 0.919 |  |
| **C19** | 0 | 0.919 | 0 | 0.919 | 0 | 0.919 | 0 | 0.919 | | 0 | 0.919 | 0 | 0.919 | 0 | | 0.919 | 0 | | 0.919 | 0 | | 0.919 |  |
| **C20** | 0 | 0.995 | 0 | 0.994 | 0 | 0.994 | 0 | 0.994 | | 0 | 0.995 | 0 | 0.995 | 0 | | 0.995 | 0 | | 0.994 | 0 | | 0.995 |  |
