# Supplementary material for: Participatory modelling for poverty alleviation using fuzzy cognitive maps and OWA learning aggregation
Source: PLoS One. 2020 Jun 8;15(6):e0233984. doi: 10.1371/journal.pone.0233984 (PMC7279611; doi:10.1371/journal.pone.0233984)
Supplement: S2 Table — (DOCX) [file pone.0233984.s007.docx]

**S2 Table. Scenario results (initial and final value) for each concept, for the Average-FCM (C)**

|  | **Scenario 1** | | **Scenario 2** | | **Scenario 3** | | **Scenario 4** | | **Scenario 5** | | | **Scenario 6** | | | **Scenario 7** | | | **Scenario 8** | | | **Scenario 9** | | |
| --- | --- | --- | --- | --- | --- | --- | --- | --- | --- | --- | --- | --- | --- | --- | --- | --- | --- | --- | --- | --- | --- | --- | --- |
| **Key Concept** | **Initial value** | **Final value** | **Initial value** | **Final value** | **Initial value** | **Final value** | **Initial value** | **Final value** | | **Initial value** | **Final value** | **Initial value** | **Final value** | **Initial value** | | **Final value** | **Initial value** | | **Final value** | **Initial value** | | **Final value** |  |
| **C1** | 1 | 1 | 0 | 0.914 | 0 | 0.882 | 0 | 0.882 | | 1 | 1 | 1 | 1 | 1 | | 1 | 0 | | 0.882 | 1 | | 1 |  |
| **C2** | 1 | 1 | 0 | 0.830 | 0 | 0.783 | 0 | 0.783 | | 1 | 1 | 1 | 1 | 1 | | 1 | 0 | | 0.783 | 1 | | 1 |  |
| **C3** | 0 | 0.659 | 1 | 1 | 0 | 0.659 | 0 | 0.659 | | 1 | 1 | 0 | 0.659 | 0 | | 0.659 | 0 | | 0.659 | 1 | | 1 |  |
| **C4** | 0 | 0.659 | 0 | 0.659 | 0 | 0.659 | 0 | 0.659 | | 0 | 0.659 | 0 | 0.659 | 0 | | 0.659 | 0 | | 0.659 | 0 | | 0.659 |  |
| **C5** | 0 | 0.769 | 0 | 0.769 | 1 | 1 | 0 | 0.769 | | 0 | 0.769 | 1 | 1 | 0 | | 0.769 | 1 | | 1 | 1 | | 1 |  |
| **C6** | 0 | 0.912 | 0 | 0.906 | 0 | 0.904 | 0 | 0.904 | | 0 | 0.912 | 0 | 0.912 | 0 | | 0.912 | 0 | | 0.904 | 0 | | 0.912 |  |
| **C7** | 0 | 0.983 | 0 | 0.982 | 0 | 0.983 | 0 | 0.985 | | 0 | 0.983 | 0 | 0.983 | 0 | | 0.985 | 0 | | 0.985 | 0 | | 0.985 |  |
| **C8** | 0 | 0.882 | 0 | 0.882 | 0 | 0.883 | 0 | 0.891 | | 0 | 0.882 | 0 | 0.883 | 0 | | 0.891 | 0 | | 0.891 | 0 | | 0.891 |  |
| **C9** | 0 | 0.873 | 0 | 0.873 | 0 | 0.889 | 1 | 1 | | 0 | 0.873 | 0 | 0.889 | 1 | | 1 | 1 | | 1 | 1 | | 1 |  |
| **C10** | 0 | 0.871 | 0 | 0.871 | 0 | 0.891 | 1 | 1 | | 0 | 0.871 | 0 | 0.891 | 1 | | 1 | 1 | | 1 | 1 | | 1 |  |
| **C11** | 0 | 0.933 | 0 | 0.933 | 0 | 0.933 | 0 | 0.933 | | 0 | 0.933 | 0 | 0.933 | 0 | | 0.933 | 0 | | 0.933 | 0 | | 0.933 |  |
| **C12** | 0 | 0.894 | 0 | 0.894 | 0 | 0.894 | 0 | 0.894 | | 0 | 0.894 | 0 | 0.894 | 0 | | 0.894 | 0 | | 0.894 | 0 | | 0.894 |  |
| **C13** | 0 | 0.659 | 0 | 0.659 | 0 | 0.659 | 0 | 0.659 | | 0 | 0.659 | 0 | 0.659 | 0 | | 0.659 | 0 | | 0.659 | 0 | | 0.659 |  |
| **C14** | 0 | 0.659 | 0 | 0.659 | 0 | 0.659 | 0 | 0.659 | | 0 | 0.659 | 0 | 0.659 | 0 | | 0.659 | 0 | | 0.659 | 0 | | 0.659 |  |
| **C15** | 0 | 0.872 | 0 | 0.872 | 0 | 0.872 | 0 | 0.872 | | 0 | 0.872 | 0 | 0.872 | 0 | | 0.872 | 0 | | 0.872 | 0 | | 0.872 |  |
| **C16** | 0 | 0.789 | 0 | 0.789 | 0 | 0.789 | 0 | 0.789 | | 0 | 0.789 | 0 | 0.789 | 0 | | 0.789 | 0 | | 0.789 | 0 | | 0.789 |  |
| **C17** | 0 | 0.889 | 0 | 0.876 | 0 | 0.890 | 0 | 0.872 | | 0 | 0.889 | 0 | 0.905 | 0 | | 0.889 | 0 | | 0.890 | 0 | | 0.905 |  |
| **C18** | 0 | 0.888 | 0 | 0.888 | 0 | 0.888 | 0 | 0.888 | | 0 | 0.888 | 0 | 0.888 | 0 | | 0.888 | 0 | | 0.888 | 0 | | 0.888 |  |
| **C19** | 0 | 0.888 | 0 | 0.888 | 0 | 0.888 | 0 | 0.888 | | 0 | 0.888 | 0 | 0.888 | 0 | | 0.888 | 0 | | 0.888 | 0 | | 0.888 |  |
| **C20** | 0 | 0.986 | 0 | 0.984 | 0 | 0.984 | 0 | 0.984 | | 0 | 0.986 | 0 | 0.986 | 0 | | 0.986 | 0 | | 0.984 | 0 | | 0.986 |  |
