# Supplementary material for: Participatory modelling for poverty alleviation using fuzzy cognitive maps and OWA learning aggregation
Source: PLoS One. 2020 Jun 8;15(6):e0233984. doi: 10.1371/journal.pone.0233984 (PMC7279611; doi:10.1371/journal.pone.0233984)
Supplement: S3 Table — (DOCX) [file pone.0233984.s008.docx]

**S3 Table. Scenario results (initial and final value) for each concept, for the OWA-FCM (L)**

|  | **Scenario 1** | | **Scenario 2** | | **Scenario 3** | | **Scenario 4** | | **Scenario 5** | | | **Scenario 6** | | | **Scenario 7** | | | **Scenario 8** | | | **Scenario 9** | | |
| --- | --- | --- | --- | --- | --- | --- | --- | --- | --- | --- | --- | --- | --- | --- | --- | --- | --- | --- | --- | --- | --- | --- | --- |
| **Key Concept** | **Initial value** | **Final value** | **Initial value** | **Final value** | **Initial value** | **Final value** | **Initial value** | **Final value** | | **Initial value** | **Final value** | **Initial value** | **Final value** | **Initial value** | | **Final value** | **Initial value** | | **Final value** | **Initial value** | | **Final value** |  |
| **C1** | 1 | 1 | 0 | 0.865 | 0 | 0.833 | 0 | 0.833 | | 1 | 1 | 1 | 1 | 1 | | 1 | 0 | | 0.833 | 1 | | 1 |  |
| **C2** | 1 | 1 | 0 | 0.802 | 0 | 0.760 | 0 | 0.760 | | 1 | 1 | 1 | 1 | 1 | | 1 | 0 | | 0.760 | 1 | | 1 |  |
| **C3** | 0 | 0.659 | 1 | 1 | 0 | 0.659 | 0 | 0.659 | | 1 | 1 | 0 | 0.659 | 0 | | 0.659 | 0 | | 0.659 | 1 | | 1 |  |
| **C4** | 0 | 0.659 | 0 | 0.659 | 0 | 0.659 | 0 | 0.659 | | 0 | 0.659 | 0 | 0.659 | 0 | | 0.659 | 0 | | 0.659 | 0 | | 0.659 |  |
| **C5** | 0 | 0.751 | 0 | 0.751 | 1 | 1 | 0 | 0.751 | | 0 | 0.751 | 1 | 1 | 0 | | 0.751 | 1 | | 1 | 1 | | 1 |  |
| **C6** | 0 | 0.878 | 0 | 0.868 | 0 | 0.865 | 0 | 0.866 | | 0 | 0.878 | 0 | 0.878 | 0 | | 0.878 | 0 | | 0.866 | 0 | | 0.878 |  |
| **C7** | 0 | 0.958 | 0 | 0.958 | 0 | 0.959 | 0 | 0.964 | | 0 | 0.958 | 0 | 0.959 | 0 | | 0.965 | 0 | | 0.964 | 0 | | 0.965 |  |
| **C8** | 0 | 0.832 | 0 | 0.832 | 0 | 0.834 | 0 | 0.845 | | 0 | 0.832 | 0 | 0.834 | 0 | | 0.845 | 0 | | 0.845 | 0 | | 0.845 |  |
| **C9** | 0 | 0.831 | 0 | 0.831 | 0 | 0.849 | 1 | 1 | | 0 | 0.831 | 0 | 0.849 | 1 | | 1 | 1 | | 1 | 1 | | 1 |  |
| **C10** | 0 | 0.824 | 0 | 0.824 | 0 | 0.845 | 1 | 1 | | 0 | 0.824 | 0 | 0.845 | 1 | | 1 | 1 | | 1 | 1 | | 1 |  |
| **C11** | 0 | 0.889 | 0 | 0.889 | 0 | 0.889 | 0 | 0.889 | | 0 | 0.889 | 0 | 0.889 | 0 | | 0.889 | 0 | | 0.889 | 0 | | 0.889 |  |
| **C12** | 0 | 0.838 | 0 | 0.838 | 0 | 0.838 | 0 | 0.838 | | 0 | 0.838 | 0 | 0.838 | 0 | | 0.838 | 0 | | 0.838 | 0 | | 0.838 |  |
| **C13** | 0 | 0.659 | 0 | 0.659 | 0 | 0.659 | 0 | 0.659 | | 0 | 0.659 | 0 | 0.659 | 0 | | 0.659 | 0 | | 0.659 | 0 | | 0.659 |  |
| **C14** | 0 | 0.659 | 0 | 0.659 | 0 | 0.659 | 0 | 0.659 | | 0 | 0.659 | 0 | 0.659 | 0 | | 0.659 | 0 | | 0.659 | 0 | | 0.659 |  |
| **C15** | 0 | 0.829 | 0 | 0.829 | 0 | 0.829 | 0 | 0.829 | | 0 | 0.829 | 0 | 0.829 | 0 | | 0.829 | 0 | | 0.829 | 0 | | 0.829 |  |
| **C16** | 0 | 0.755 | 0 | 0.755 | 0 | 0.755 | 0 | 0.755 | | 0 | 0.755 | 0 | 0.755 | 0 | | 0.755 | 0 | | 0.755 | 0 | | 0.755 |  |
| **C17** | 0 | 0.845 | 0 | 0.829 | 0 | 0.844 | 0 | 0.826 | | 0 | 0.845 | 0 | 0.862 | 0 | | 0.845 | 0 | | 0.844 | 0 | | 0.862 |  |
| **C18** | 0 | 0.838 | 0 | 0.838 | 0 | 0.838 | 0 | 0.838 | | 0 | 0.838 | 0 | 0.838 | 0 | | 0.838 | 0 | | 0.838 | 0 | | 0.838 |  |
| **C19** | 0 | 0.848 | 0 | 0.848 | 0 | 0.848 | 0 | 0.848 | | 0 | 0.848 | 0 | 0.848 | 0 | | 0.848 | 0 | | 0.848 | 0 | | 0.848 |  |
| **C20** | 0 | 0.969 | 0 | 0.966 | 0 | 0.965 | 0 | 0.965 | | 0 | 0.969 | 0 | 0.970 | 0 | | 0.969 | 0 | | 0.966 | 0 | | 0.970 |  |
