# Supplementary material for: Participatory modelling for poverty alleviation using fuzzy cognitive maps and OWA learning aggregation
Source: PLoS One. 2020 Jun 8;15(6):e0233984. doi: 10.1371/journal.pone.0233984 (PMC7279611; doi:10.1371/journal.pone.0233984)
Supplement: S4 Table — (DOCX) [file pone.0233984.s009.docx]

**S9 Table. Scenario results (initial and final value) for each concept, for the OWA-FCM (C)**

|  | **Scenario 1** | | **Scenario 2** | | **Scenario 3** | | **Scenario 4** | | **Scenario 5** | | | **Scenario 6** | | | **Scenario 7** | | | **Scenario 8** | | | **Scenario 9** | | |
| --- | --- | --- | --- | --- | --- | --- | --- | --- | --- | --- | --- | --- | --- | --- | --- | --- | --- | --- | --- | --- | --- | --- | --- |
| **Key Concept** | **Initial value** | **Final value** | **Initial value** | **Final value** | **Initial value** | **Final value** | **Initial value** | **Final value** | | **Initial value** | **Final value** | **Initial value** | **Final value** | **Initial value** | | **Final value** | **Initial value** | | **Final value** | **Initial value** | | **Final value** |  |
| **C1** | 1 | 1 | 0 | 0.816 | 0 | 0.787 | 0 | 0.787 | | 1 | 1 | 1 | 1 | 1 | | 1 | 0 | | 0.787 | 1 | | 1 |  |
| **C2** | 1 | 1 | 0 | 0.759 | 0 | 0.728 | 0 | 0.728 | | 1 | 1 | 1 | 1 | 1 | | 1 | 0 | | 0.728 | 1 | | 1 |  |
| **C3** | 0 | 0.659 | 1 | 1 | 0 | 0.659 | 0 | 0.659 | | 1 | 1 | 0 | 0.659 | 0 | | 0.659 | 0 | | 0.659 | 1 | | 1 |  |
| **C4** | 0 | 0.659 | 0 | 0.659 | 0 | 0.659 | 0 | 0.659 | | 0 | 0.659 | 0 | 0.659 | 0 | | 0.659 | 0 | | 0.659 | 0 | | 0.659 |  |
| **C5** | 0 | 0.711 | 0 | 0.711 | 1 | 1 | 0 | 0.711 | | 0 | 0.711 | 1 | 1 | 0 | | 0.711 | 1 | | 1 | 1 | | 1 |  |
| **C6** | 0 | 0.810 | 0 | 0.798 | 0 | 0.796 | 0 | 0.797 | | 0 | 0.810 | 0 | 0.810 | 0 | | 0.811 | 0 | | 0.797 | 0 | | 0.811 |  |
| **C7** | 0 | 0.887 | 0 | 0.886 | 0 | 0.887 | 0 | 0.900 | | 0 | 0.887 | 0 | 0.888 | 0 | | 0.900 | 0 | | 0.900 | 0 | | 0.900 |  |
| **C8** | 0 | 0.763 | 0 | 0.763 | 0 | 0.764 | 0 | 0.778 | | 0 | 0.763 | 0 | 0.764 | 0 | | 0.778 | 0 | | 0.778 | 0 | | 0.778 |  |
| **C9** | 0 | 0.770 | 0 | 0.770 | 0 | 0.789 | 1 | 1 | | 0 | 0.770 | 0 | 0.789 | 1 | | 1 | 1 | | 1 | 1 | | 1 |  |
| **C10** | 0 | 0.753 | 0 | 0.753 | 0 | 0.771 | 1 | 1 | | 0 | 0.753 | 0 | 0.771 | 1 | | 1 | 1 | | 1 | 1 | | 1 |  |
| **C11** | 0 | 0.829 | 0 | 0.829 | 0 | 0.829 | 0 | 0.83 | | 0 | 0.829 | 0 | 0.829 | 0 | | 0.83 | 0 | | 0.83 | 0 | | 0.83 |  |
| **C12** | 0 | 0.778 | 0 | 0.778 | 0 | 0.778 | 0 | 0.778 | | 0 | 0.778 | 0 | 0.778 | 0 | | 0.778 | 0 | | 0.778 | 0 | | 0.778 |  |
| **C13** | 0 | 0.659 | 0 | 0.659 | 0 | 0.659 | 0 | 0.659 | | 0 | 0.659 | 0 | 0.659 | 0 | | 0.659 | 0 | | 0.659 | 0 | | 0.659 |  |
| **C14** | 0 | 0.659 | 0 | 0.659 | 0 | 0.659 | 0 | 0.659 | | 0 | 0.659 | 0 | 0.659 | 0 | | 0.659 | 0 | | 0.659 | 0 | | 0.659 |  |
| **C15** | 0 | 0.761 | 0 | 0.761 | 0 | 0.761 | 0 | 0.761 | | 0 | 0.761 | 0 | 0.761 | 0 | | 0.761 | 0 | | 0.761 | 0 | | 0.761 |  |
| **C16** | 0 | 0.717 | 0 | 0.717 | 0 | 0.717 | 0 | 0.717 | | 0 | 0.717 | 0 | 0.717 | 0 | | 0.717 | 0 | | 0.717 | 0 | | 0.717 |  |
| **C17** | 0 | 0.791 | 0 | 0.775 | 0 | 0.791 | 0 | 0.773 | | 0 | 0.791 | 0 | 0.808 | 0 | | 0.791 | 0 | | 0.791 | 0 | | 0.808 |  |
| **C18** | 0 | 0.771 | 0 | 0.771 | 0 | 0.771 | 0 | 0.771 | | 0 | 0.771 | 0 | 0.771 | 0 | | 0.771 | 0 | | 0.771 | 0 | | 0.771 |  |
| **C19** | 0 | 0.778 | 0 | 0.778 | 0 | 0.778 | 0 | 0.778 | | 0 | 0.778 | 0 | 0.778 | 0 | | 0.778 | 0 | | 0.778 | 0 | | 0.778 |  |
| **C20** | 0 | 0.873 | 0 | 0.866 | 0 | 0.866 | 0 | 0.866 | | 0 | 0.873 | 0 | 0.874 | 0 | | 0.874 | 0 | | 0.866 | 0 | | 0.874 |  |
