# Supplementary material for: Participatory modelling for poverty alleviation using fuzzy cognitive maps and OWA learning aggregation
Source: PLoS One. 2020 Jun 8;15(6):e0233984. doi: 10.1371/journal.pone.0233984 (PMC7279611; doi:10.1371/journal.pone.0233984)
Supplement: Attachment — Submitted filename: Response to Reviewers-Final.docx [file pone.0233984.s010.docx]

**Authors’ Responses to Comments by the Academic Editor**

| **Sr** | **Comments by the Academic Editor** | **Responses of Authors** |
| --- | --- | --- |
| 1 | Please include the following items when submitting your revised manuscript:   - A rebuttal letter that responds to each point raised by the academic editor and reviewer(s). This letter should be uploaded as separate file and labeled 'Response to Reviewers'. - A marked-up copy of your manuscript that highlights changes made to the original version. This file should be uploaded as separate file and labeled 'Revised Manuscript with Track Changes'. - An unmarked version of your revised paper without tracked changes. This file should be uploaded as separate file and labeled 'Manuscript'. | The authors have included all the required items with the revised manuscript. |
| 2 | Please ensure that your manuscript meets PLOS ONE's style requirements, including those for file naming. The PLOS ONE style templates can be found at  <http://www.journals.plos.org/plosone/s/file?id=wjVg/PLOSOne_formatting_sample_main_body.pdf> and <http://www.journals.plos.org/plosone/s/file?id=ba62/PLOSOne_formatting_sample_title_authors_affiliations.pdf> | The authors have ensured that the manuscript meets PLOS ONE's style requirements, including those for file naming. |
| 3 | We note that Figures 1, 7 and 8 in your submission contain copyrighted images. All PLOS content is published under the Creative Commons Attribution License (CC BY 4.0), which means that the manuscript, images, and Supporting Information files will be freely available online, and any third party is permitted to access, download, copy, distribute, and use these materials in any way, even commercially, with proper attribution. For more information, see our copyright guidelines: <http://journals.plos.org/plosone/s/licenses-and-copyright>.  We require you to either (1) present written permission from the copyright holder to publish these figures specifically under the CC BY 4.0 license, or (2) remove the figures from your submission:  I. You may seek permission from the original copyright holder of Figures 1, 7 and 8 to publish the content specifically under the CC BY 4.0 license.  We recommend that you contact the original copyright holder with the Content Permission Form (<http://journals.plos.org/plosone/s/file?id=7c09/content-permission-form.pdf>) and the following text:  “I request permission for the open-access journal PLOS ONE to publish XXX under the Creative Commons Attribution License (CCAL) CC BY 4.0 (<http://creativecommons.org/licenses/by/4.0/>). Please be aware that this license allows unrestricted use and distribution, even commercially, by third parties. Please reply and provide explicit written permission to publish XXX under a CC BY license and complete the attached form.”  Please upload the completed Content Permission Form or other proof of granted permissions as an "Other" file with your submission.  In the figure caption of the copyrighted figure, please include the following text: “Reprinted from [ref] under a CC BY license, with permission from [name of publisher], original copyright [original copyright year].”  II. If you are unable to obtain permission from the original copyright holder to publish these figures under the CC BY 4.0 license or if the copyright holder’s requirements are incompatible with the CC BY 4.0 license, please either i) remove the figure or ii) supply a replacement figure that complies with the CC BY 4.0 license. Please check copyright information on all replacement figures and update the figure caption with source information. If applicable, please specify in the figure caption text when a figure is similar but not identical to the original image and is therefore for illustrative purposes only. | Figures 7 and 8 have been removed in the revised manuscript. They are outcomes of a java-based tool that has been developed by PhD student, for research purposes and no software license has been attributed yet.  In Figure 1, we had inserted SDG1 graphics, which is removed in the new Figure in the revised manuscript. Since we earlier worked on the national evaluation of DAY-NRLM, the Theory of Change for DAY-NRLM, which we had developed in consultation with the programme implementers, had some pictorial designs. We developed the Theory of Change as depicted in Figure 1 of this paper in consultation with the programme implementers (Jammu and Kashmir State Rural Livelihood Mission). All the pictorial graphics are removed in the current version of Figure 1. This Figure is similar but not identical to the one developed for the national DAY-NRLM. |
| 4 | Thank you for stating the following in the Acknowledgments Section of your manuscript:  "Part of this work has received funding from the Ministry of Rural Development, Government of India for providing financial support."  We note that you have provided funding information that is not currently declared in your Funding Statement. However, funding information should not appear in the Acknowledgments section or other areas of your manuscript. We will only publish funding information present in the Funding Statement section of the online submission form.  Please remove any funding-related text from the manuscript and let us know how you would like to update your Funding Statement. Currently, your Funding Statement reads as follows:  The author(s) received no specific funding for this work. | Funding related text is deleted from the manuscript.  The Funding Disclosure Statement is updated. |
| 5 | Please ensure that you refer to Figure 2 in your text as, if accepted, production will need this reference to link the reader to the figure. | The authors have provided the reference to Figure 2 in the text in the revised manuscript. |

**Reviewer's Responses to Questions**

| **Sr** | **Questions** | **Reviewer #1** | **Reviewer #2** | **Reviewer #3** | **Authors’ Responses** |
| --- | --- | --- | --- | --- | --- |
| 1 | Is the manuscript technically sound, and do the data support the conclusions? | Partly | Yes | Yes |  |
| 2 | Has the statistical analysis been performed appropriately and rigorously? | I Don't Know | Yes | Yes |  |
| 3 | Have the authors made all data underlying the findings in their manuscript fully available? | Yes | Yes | Yes |  |
| 4 | Is the manuscript presented in an intelligible fashion and written in standard English? | Yes | Yes | Yes | Extensive editing of the English language is made in this manuscript. |

**5. Review Comments to Authors**

**Authors’ Responses to Comments by Reviewers**

**Reviewer #1:**

| **Sr** | **Comments by Reviewer #1** | **Authors’ Responses** |
| --- | --- | --- |
| 1 | (No comments) | - We thank the reviewer for approving the manuscript for publication fully. |

**Reviewer #2:**

| **Sr** | **Comments by Reviewer #2** | **Authors’ Responses** |
| --- | --- | --- |
| 1 | The article title is appropriate.  The abstract accurately reflects the content.  The primary objective is genuine and argued persuasively.  The purported significance of the article is explicitly stated.  The article adequately ties to the relevant literature.  The research study methods are sound and appropriate.  The writing is clear, concise and interesting however further proofreading should be done to correct some typographical mistakes. | - We thank you very much for your appreciation and insightful comments, which helped improve the paper. We have tried to address all your concerns. |
| 2 | Also, the author should check the Sentence in line numbers 253-255 (i.e. Each adjacency matrix consists of the normalised………………was called FCM (L) model) and correct the grammatical error therein. | - This is corrected in the revised version of the manuscript. |
| 3 | All figures, tables, and images are necessary and appropriate.  The conclusions are accurate and supported by the content.  The article has made sufficient contribution to knowledge  Not all articles in reference are cited. Authors should delete references not cited in the manuscript. | - The list of references have been updated and correctly cited in the revised version of the manuscript. |

**Reviewer #3:**

| **Sr** | **Comments by Reviewer #3** | **Authors’ Responses** |
| --- | --- | --- |
| 1 | The paper is well written but there are some points: | - We welcome and appreciate your insightful comments, which helped improving the paper. We have tried to address the points raised in the revised manuscript. |
| 2 | Figures are not clear at all. | - All the Figures in this study are created in the best possible resolution and were uploaded individually according to the journal’s guidelines. However, during the initial submission, the Figures were somehow distorted after being zoomed in and transformed to pdf which was automatically created by the system. |
| 3 | It is recommended to compare this proposed model with new developed FCMs such Neuro-FCM (NFCM). | - In response to the Reviewer’s comment, the authors added this reference in the introduction section, with a brief discussion about NFCM and its capabilities in decision-making tasks. - In particular, for the construction of the NFCM, the weights of connecting links are determined by using a neuro-fuzzy system (NFIS). NFIS approach needs empirical data in the form of fuzzy rules to define the weights of the model. By using the known weight values, the network is trained until the desired values of NFIS parameters are obtained. - The most important issue in designing an NFCM model, as described in Amirkhani, et al. (2019), is the construction of an appropriate set of fuzzy rules defined by experts for this model and the calculation of model weights through training. - Thus, it is evident that NFCM uses empirical data to build a neuro-fuzzy system for modeling the inherent fuzziness that exists in them. - The authors of this study didn't deal with empirical data (fuzzy rules) as needed for NFCM construction and presented in the work of Amirkhani, et al. (2019). Instead, we followed a specific protocol using stakeholders’ perceptions to design the FCM model by assigning numerical values to weighted interconnections among concepts for policy-making purposes in the domain of socio-economic poverty alleviation of poor rural communities. - Thus, it is not feasible to provide a straightforward comparison with NFCM, and we are stack to this common FCM design process. A simple reference though, on the NFCM and its applicability under certain conditions, is desired. |
| 4 | It is expected to proof the performance of proposed model in mathematical manner not just giving an example. | - Authors do not need to prove the implemented approach mathematically as this is exclusively based on a previous approach, which has been proven and presented in Papageorgiou, et al. (2020) and dealt with the OWA aggregation method and especially for learning OWA operators. Specifically, in that work, a step by step application of the learning OWA operators for defining the OWA FCM weights was presented, in a simple FCM that was constructed by the experts. - The proposed model was accordingly adapted to the context of this study and particularly for the aggregation of FCM weights. Thus, there was no need to present it further and again, mathematically prove its performance. - For the convenience of the Reviewers, the authors cite their previous work in “Sustainability”, where a complete example is presented Papageorgiou, et al. (2020). |
