# Supplementary material for: Participatory modelling for poverty alleviation using fuzzy cognitive maps and OWA learning aggregation
Source: PLoS One. 2020 Jun 8;15(6):e0233984. doi: 10.1371/journal.pone.0233984 (PMC7279611; doi:10.1371/journal.pone.0233984)
Supplement: Attachment — Submitted filename: Response to Reviewers.docx [file pone.0233984.s012.docx]

**Second Revision:**

**Reviewer's Responses to Questions**

| **Sr** | **Questions** | **Reviewer #1** | **Reviewer #2** | **Reviewer #3** | **Authors’ Responses** |
| --- | --- | --- | --- | --- | --- |
| 1 | If the authors have adequately addressed your comments raised in a previous round of review and you feel that this manuscript is now acceptable for publication, you may indicate that here to bypass the “Comments to the Author” section, enter your conflict of interest statement in the “Confidential to Editor” section, and submit your "Accept" recommendation. | (No Response) | All comments have been addressed | All comments have been addressed |  |
| 2 | Is the manuscript technically sound, and do the data support the conclusions? | Yes | Yes | Yes |  |
| 3 | Has the statistical analysis been performed appropriately and rigorously? | Yes | Yes | I Don't Know |  |
| 4 | Have the authors made all data underlying the findings in their manuscript fully available? | Yes | No | (No Response) |  |
| 5 | Is the manuscript presented in an intelligible fashion and written in standard English? | Yes | Yes | Yes |  |

**6. Review Comments to Authors**

**Authors’ Responses to Comments by Reviewers**

**Reviewer #1:**

| **Sr** | **Comments by Reviewer #1** | **Authors’ Responses** |
| --- | --- | --- |
| 1 | The manuscript Overall is well structured and well organised.  It also contributes to the emerging FCM-based scenario domains. However, I have some minor and a few major concerns about some parts of the context.  I understand that paragraph four (A new extension of FCM, known as NFCM … experts and the calculation of model weights through training.) was later provided in the revision version in response to a comment by a reviewer.  However, it appears entirely disengaged from the main topic while adding no value to the text. I would suggest moving this paragraph to the  ‘Discussion’ section, which can be further expanded by analysing the similarities, differences, advantages and possible disadvantages of each technique. This modification will be in turn in line with the  ‘Discussion’ context. | - We thank you very much for your appreciation and insightful comments, which helped improve the paper. We have tried to address all your concerns. - Thank you for the comment concerning this paragraph. We have significantly shortened it, keeping only the most important key issues. Thus, we believe that this paragraph will be well engaged with the content of this section and does not need to be transferred to another section. - Also, we reduced the length of certain sections, like the “introduction” and “methodology”, shortening the overall length of the revised manuscript, according to Reviewers’ concerns, without omitting the most significant issues. |
| 2 | Section one (1. Introduction) is simply too long. The subsection 1.1 (Theory of change for decreased socio-economic poverty in Jammu and Kashmir) while being too long in the title, can come separately and independently as a case study section, perhaps after the methodology section. This subsection indeed comes between two main parts of the section, the first introducing the research, and the second providing the contributions of the paper along with the summarisation of the paper structure. | The sub-title is shortened.  The placing of this sub-section is appropriate as the theory of change helps us to develop input vectors for policy scenarios. In methodology section, the rationale for deciding input vectors will not be clear unless it is discussed before the methodology section. Instead of keeping under methodology section, we prefer to place it before. |
| 3 | In section 2.2.1 (Expert-based FCM using an open-concept design) who are those 31 experts (their job title, position, their field, etc.)? How many workshops were arranged? | During expert-based FCM using an open-concept design we had one workshop participated by 31 national and state-level implementers of the DAY-NRLM programme. The participants constituted 6 generalists (in-charge of administration of the programme) and 22 specialists in the area of community development, micro-finance, farm and non-farm livelihoods, and monitoring and evaluation including three experts who were involved in designing the programme. We classified all of them as implementers of the programme. |
| 4 | Line 360, I guess the correct number for the figure caption “Fig 3a. Collective-FCM model for Average-FCM (L)” is Fig. 5a. Sub-titles are often too long. When possible, please shorten by keeping only the keywords. | - In the previously revised manuscript, the figure caption was correctly written as Fig 5a and not as Fig 3a. - Figures captions have been shortened, where needed. |
| 5 | In the text, ‘Figure’ sometimes come as abbreviated ‘Fig’, and sometimes come fully ‘Figure’. The label should be consistent through the whole text. The order of figures at the end is pretty sloppy. It starts with ‘Fig. 2’-‘Fig. 14’ and then ends with ‘Fig. 1’ and ‘Fig. 3’. | - The word “Figure” was replaced by the abbreviation “Fig” throughout the revised manuscript, following your suggestion. - Authors checked carefully the order of all figures in the manuscript and amended or deleted the parts that possibly caused confusion to readers. |
| 6 | Section 2 is again too long and contains arguably too many subsections. Breaking down this section into two sections could improve the structure of the work. | - Thank you for your comment. Section 2 has been split into two new sections. Section 2 of the revised manuscript presents the methodology for FCM development, whereas section 3 contains the scenario analysis and simulation process. |
| 7 | Figures 7&8 can be moved to the supplementary information as they do not offer many contributions to the richness of the text body. Discussion can come as an independent section, and it is better to come after ‘Policy implications of the scenario results’ to keep the coherency. | - Thank you for the comment. Figures 7 and 8 have been moved to the supplementary information as S4 and S5 Figs. - Following your suggestion, we also transferred the “Discussion” section after “Policy implications of the scenario results”. |
| 8 | In section 2.8 (Structural analysis), the paper promises providing indices such as complexity, density, and the hierarchy index, but they were never brought. | - In the revised version of the manuscript, we added the values of these structural analysis indices (L351-L353), as requested by the Reviewer. |

**Reviewer #2:**

| **Sr** | **Comments by Reviewer #2** | **Authors’ Responses** |
| --- | --- | --- |
| 1 | The abstract is good. The primary objective is good.  Introduction: This is good and comprehensive. It aligns with the research objective. However, it is too long. I advise the author to reduce the length of the introduction. The key issues should be focused on.  The Literature reviewed is okay. | - We thank you very much for your appreciation and insightful comments, which helped improve the paper. We have tried to address all your concerns. - Authors revised the “Introduction” section by reducing its length, while trying to keep the key issues. |
| 2 | Methodology: This is good and well directed towards achieving the aim and objective of the research. However, I advise the author to reduce the length and hence focus on only the most important concepts adopted and the model developed and the implementation.  The figures and graphs are okay.  The conclusion is okay. | - Thank you for the comment. Methodology (section 2) has been broken down into two main sections, as it was too long and difficult to follow. In the revised manuscript, section 2 presents the methodology for FCM development, whereas section 3 contains the scenario analysis and simulation process. |
| 3 | General Comments: The manuscript is too long. The author should focus only on key concepts relevant to the model development and implementation with view to reduce the length of the manuscript. | - Thank you for the comment. The length of the manuscript has been reduced adequately to meet Reviewer’s concerns, keeping only the most important key issues. More specifically, we reduced the length of certain sections, like the “introduction” and “methodology”, shortening the overall length of the revised manuscript. The version with the track-changes will provide a clearer view of the changes that have been made. |

**Reviewer #3:**

| **Sr** | **Comments by Reviewer #3** | **Authors’ Responses** |
| --- | --- | --- |
| 1 | It is well written and all the comments are addressed. Now it is ready for publication. | - We thank the reviewer for approving the manuscript for publication. |

**First Revision:**

**Authors’ Responses to Comments by the Academic Editor**

| **Sr** | **Comments by the Academic Editor** | **Responses of Authors** |
| --- | --- | --- |
| 1 | Please include the following items when submitting your revised manuscript:   - A rebuttal letter that responds to each point raised by the academic editor and reviewer(s). This letter should be uploaded as separate file and labeled 'Response to Reviewers'. - A marked-up copy of your manuscript that highlights changes made to the original version. This file should be uploaded as separate file and labeled 'Revised Manuscript with Track Changes'. - An unmarked version of your revised paper without tracked changes. This file should be uploaded as separate file and labeled 'Manuscript'. | The authors have included all the required items with the revised manuscript. |
| 2 | Please ensure that your manuscript meets PLOS ONE's style requirements, including those for file naming. The PLOS ONE style templates can be found at  <http://www.journals.plos.org/plosone/s/file?id=wjVg/PLOSOne_formatting_sample_main_body.pdf> and <http://www.journals.plos.org/plosone/s/file?id=ba62/PLOSOne_formatting_sample_title_authors_affiliations.pdf> | The authors have ensured that the manuscript meets PLOS ONE's style requirements, including those for file naming. |
| 3 | We note that Figures 1, 7 and 8 in your submission contain copyrighted images. All PLOS content is published under the Creative Commons Attribution License (CC BY 4.0), which means that the manuscript, images, and Supporting Information files will be freely available online, and any third party is permitted to access, download, copy, distribute, and use these materials in any way, even commercially, with proper attribution. For more information, see our copyright guidelines: <http://journals.plos.org/plosone/s/licenses-and-copyright>.  We require you to either (1) present written permission from the copyright holder to publish these figures specifically under the CC BY 4.0 license, or (2) remove the figures from your submission:  I. You may seek permission from the original copyright holder of Figures 1, 7 and 8 to publish the content specifically under the CC BY 4.0 license.  We recommend that you contact the original copyright holder with the Content Permission Form (<http://journals.plos.org/plosone/s/file?id=7c09/content-permission-form.pdf>) and the following text:  “I request permission for the open-access journal PLOS ONE to publish XXX under the Creative Commons Attribution License (CCAL) CC BY 4.0 (<http://creativecommons.org/licenses/by/4.0/>). Please be aware that this license allows unrestricted use and distribution, even commercially, by third parties. Please reply and provide explicit written permission to publish XXX under a CC BY license and complete the attached form.”  Please upload the completed Content Permission Form or other proof of granted permissions as an "Other" file with your submission.  In the figure caption of the copyrighted figure, please include the following text: “Reprinted from [ref] under a CC BY license, with permission from [name of publisher], original copyright [original copyright year].”  II. If you are unable to obtain permission from the original copyright holder to publish these figures under the CC BY 4.0 license or if the copyright holder’s requirements are incompatible with the CC BY 4.0 license, please either i) remove the figure or ii) supply a replacement figure that complies with the CC BY 4.0 license. Please check copyright information on all replacement figures and update the figure caption with source information. If applicable, please specify in the figure caption text when a figure is similar but not identical to the original image and is therefore for illustrative purposes only. | Figures 7 and 8 have been removed in the revised manuscript.  In Figure 1, we had inserted SDG1 graphics, which is removed in the new Figure in the revised manuscript. Since we earlier worked on the national evaluation of DAY-NRLM, the Theory of Change for DAY-NRLM, which we had developed in consultation with the programme implementers, had some pictorial designs. We developed the Theory of Change as depicted in Figure 1 of this paper in consultation with the programme implementers (Jammu and Kashmir State Rural Livelihood Mission). All the pictorial graphics are removed in the current version of Figure 1. This Figure is similar but not identical to the one developed for the national DAY-NRLM. |
| 4 | Thank you for stating the following in the Acknowledgments Section of your manuscript:  "Part of this work has received funding from the Ministry of Rural Development, Government of India for providing financial support."  We note that you have provided funding information that is not currently declared in your Funding Statement. However, funding information should not appear in the Acknowledgments section or other areas of your manuscript. We will only publish funding information present in the Funding Statement section of the online submission form.  Please remove any funding-related text from the manuscript and let us know how you would like to update your Funding Statement. Currently, your Funding Statement reads as follows:  The author(s) received no specific funding for this work. | Funding related text is deleted from the manuscript.  The Funding Disclosure Statement is updated. |
| 5 | Please ensure that you refer to Figure 2 in your text as, if accepted, production will need this reference to link the reader to the figure. | The authors have provided the reference to Figure 2 in the text in the revised manuscript. |

**Reviewer's Responses to Questions**

| **Sr** | **Questions** | **Reviewer #1** | **Reviewer #2** | **Reviewer #3** | **Authors’ Responses** |
| --- | --- | --- | --- | --- | --- |
| 1 | Is the manuscript technically sound, and do the data support the conclusions? | Partly | Yes | Yes |  |
| 2 | Has the statistical analysis been performed appropriately and rigorously? | I Don't Know | Yes | Yes |  |
| 3 | Have the authors made all data underlying the findings in their manuscript fully available? | Yes | Yes | Yes |  |
| 4 | Is the manuscript presented in an intelligible fashion and written in standard English? | Yes | Yes | Yes | Extensive editing of the English language is made in this manuscript. |

**5. Review Comments to Authors**

**Authors’ Responses to Comments by Reviewers**

**Reviewer #1:**

| **Sr** | **Comments by Reviewer #1** | **Authors’ Responses** |
| --- | --- | --- |
| 1 | (No comments) | - We thank the reviewer for approving the manuscript for publication fully. |

**Reviewer #2:**

| **Sr** | **Comments by Reviewer #2** | **Authors’ Responses** |
| --- | --- | --- |
| 1 | The article title is appropriate.  The abstract accurately reflects the content.  The primary objective is genuine and argued persuasively. The purported significance of the article is explicitly stated. The article adequately ties to the relevant literature. The research study methods are sound and appropriate. The writing is clear, concise and interesting however further proofreading should be done to correct some typographical mistakes. | - We thank you very much for your appreciation and insightful comments, which helped improve the paper. We have tried to address all your concerns. |
| 2 | Also, the author should check the Sentence in line numbers 253-255 (i.e. Each adjacency matrix consists of the normalised ……………… was called FCM (L) model) and correct the grammatical error therein. | - This is corrected in the revised version of the manuscript. |
| 3 | All figures, tables, and images are necessary and appropriate. The conclusions are accurate and supported by the content. The article has made sufficient contribution to knowledge. Not all articles in reference are cited. Authors should delete references not cited in the manuscript. | - The list of references have been updated and correctly cited in the revised version of the manuscript. |

**Reviewer #3:**

| **Sr** | **Comments by Reviewer #3** | **Authors’ Responses** |
| --- | --- | --- |
| 1 | The paper is well written but there are some points: | - We welcome and appreciate your insightful comments, which helped improving the paper. We have tried to address the points raised in the revised manuscript. |
| 2 | Figures are not clear at all. | - All the Figures in this study are created in the best possible resolution and were uploaded individually according to the journal’s guidelines. However, during the initial submission, the Figures were somehow distorted after being zoomed in and transformed to pdf which was automatically created by the system. |
| 3 | It is recommended to compare this proposed model with new developed FCMs such Neuro-FCM (NFCM). | - In response to the Reviewer’s comment, the authors added this reference in the introduction section, with a brief discussion about NFCM and its capabilities in decision-making tasks. - In particular, for the construction of the NFCM, the weights of connecting links are determined by using a neuro-fuzzy system (NFIS). NFIS approach needs empirical data in the form of fuzzy rules to define the weights of the model. By using the known weight values, the network is trained until the desired values of NFIS parameters are obtained. - The most important issue in designing an NFCM model, as described in Amirkhani, et al. (2019), is the construction of an appropriate set of fuzzy rules defined by experts for this model and the calculation of model weights through training. - Thus, it is evident that NFCM uses empirical data to build a neuro-fuzzy system for modeling the inherent fuzziness that exists in them. - The authors of this study didn't deal with empirical data (fuzzy rules) as needed for NFCM construction and presented in the work of Amirkhani, et al. (2019). Instead, we followed a specific protocol using stakeholders’ perceptions to design the FCM model by assigning numerical values to weighted interconnections among concepts for policy-making purposes in the domain of socio-economic poverty alleviation of poor rural communities. - Thus, it is not feasible to provide a straightforward comparison with NFCM, and we are stack to this common FCM design process. A simple reference though, on the NFCM and its applicability under certain conditions, is desired. |
| 4 | It is expected to proof the performance of proposed model in mathematical manner not just giving an example. | - Authors do not need to prove the implemented approach mathematically as this is exclusively based on a previous approach, which has been proven and presented in Papageorgiou, et al. (2020) and dealt with the OWA aggregation method and especially for learning OWA operators. Specifically, in that work, a step by step application of the learning OWA operators for defining the OWA FCM weights was presented, in a simple FCM that was constructed by the experts. - The proposed model was accordingly adapted to the context of this study and particularly for the aggregation of FCM weights. Thus, there was no need to present it further and again, mathematically prove its performance. - For the convenience of the Reviewers, the authors cite their previous work in “Sustainability”, where a complete example is presented Papageorgiou, et al. (2020). |
