# Supplementary material for: Participatory modelling for poverty alleviation using fuzzy cognitive maps and OWA learning aggregation
Source: PLoS One. 2020 Jun 8;15(6):e0233984. doi: 10.1371/journal.pone.0233984 (PMC7279611; doi:10.1371/journal.pone.0233984)
Supplement: Attachment — Submitted filename: Reviewer comments-PONE-D-19-35078R1.pdf [file pone.0233984.s011.pdf]

PONE-D-19-35078R1
Participatory modelling for poverty alleviation using fuzzy cognitive maps and OWA learning aggregation
PLOS ONE

**COMMENTS**

1. Recommendation (Accept, Minor Revision, Major Revision, Reject) – **Accept**

2. Is the manuscript technically sound, and do the data support the conclusions? (Answer options: Yes, No, Partly) - **Yes**

3. Has the statistical analysis been performed appropriately and rigorously? (Answer options: Yes, No, I don't know, N/A) - **Yes**

4. Does the manuscript adhere to the PLOS Data Policy? Additional details can be found at http://www.plosone.org/static/policies#sharing - **Yes**

5. Is the manuscript presented in an intelligible fashion and written in standard English? (Answer options: Yes, No) - **Yes**

6. Review Comments to the Author - Below are my comments

The abstract is good. The primary objective is good.

**Introduction:** This is good and comprehensive. It aligns with the research objective. However, it is too long. I advise the author to reduce the length of the introduction. The key issues should be focused on.

The Literature reviewed is okay.

**Methodology:** This is good and well directed towards achieving the aim and objective of the research. However, I advise the author to reduce the length and hence focus on only the most important concepts adopted and the model developed and the implementation.

The figures and graphs are okay.

The conclusion is okay.

**General Comments:** The manuscript is too long. The author should focus only on key concepts relevant to the model development and implementation with view to reduce the length of the manuscript.

7. Would you like your identity revealed to the authors of this submission? (Answer options: Yes, No) - **No**

8. Do you have any potentially competing interests? If none, type "None." Our policy on competing interests can be found at http://www.plosone.org/static/policies.action#competing. - **None**
